# Deep learning for diagnosis of malign pleural effusion on computed tomography images

**DOI:** 10.1016/j.clinsp.2023.100210

**Published:** 2023-05-05

**Authors:** Neslihan Ozcelik, Ali Erdem Ozcelik, Nese Merve Guner Zirih, Inci Selimoglu, Aziz Gumus

**Affiliations:** a Recep Tayyip Erdogan University, Faculty of Medicine, Training and Research Hospital, Chest Disease, Rize, Turkey; b Recep Tayyip Erdogan University, Engineering and Architecture Faculty, Department of Landscape Architecture (Geomatics Engineer), Rize, Turkey

**Keywords:** Lung cancer, Deep learning, Pleural effusion, Decision support system, Artificial intelligence

## Abstract

**Background:**

The pleura is a serous membrane that surrounds the lungs. The visceral surface secretes fluid into the serous cavity and the parietal surface ensures a regular absorption of this fluid. If this balance is disturbed, fluid accumulation occurs in the pleural space called “Pleural Effusion”. Today, accurate diagnosis of pleural diseases is becoming more critical, as advances in treatment protocols have contributed positively to prognosis. Our aim is to perform computer-aided numerical analysis of Computed Tomography (CT) images from patients showing pleural effusion images on CT and to examine the prediction of malignant/benign distinction using deep learning by comparing with the cytology results.

**Methods:**

The authors classified 408 CT images from 64 patients whose etiology of pleural effusion was investigated using the deep learning method. 378 of the images were used for the training of the system; 15 malignant and 15 benign CT images, which were not included in the training group, were used as the test.

**Results:**

Among the 30 test images evaluated in the system; 14 of 15 malignant patients and 13 of 15 benign patients were estimated with correct diagnosis (PPD: 93.3%, NPD: 86.67%, Sensitivity: 87.5%, Specificity: 92.86%).

**Conclusion:**

Advances in computer-aided diagnostic analysis of CT images and obtaining a pre-diagnosis of pleural fluid may reduce the need for interventional procedures by guiding physicians about which patients may have malignancies. Thus, it is cost and time-saving in patient management, allowing earlier diagnosis and treatment.

## Highlights

• Decision support systems that can predict the diagnosis of malignancy for pleural effusion will help physicians in patient management.
• Advances in computer-aided diagnostic analysis of CT images and obtaining a pre-diagnosis of pleural fluid may reduce the need for interventional procedures by guiding physicians about which patients may have malignancy.
• Image-based decision support systems enable early diagnosis and treatment by saving cost and time in patient management.

## Introduction

Pleural diseases and Pleural Effusion (PE) usually occur due to secondary causes affecting the pleura. Pneumonia, heart failure, pulmonary embolism, and malignancies are the most common diseases that cause effusion.^1^ Rarely, it may develop primarily due to local effects of the pleura or mesothelioma, a primary malignant tumor. In general, PE affects more than 0.3% of the world population in each year.^2^ The incidence of pleural effusion is 3–5/1000 person-years in the literature.^3^ In addition to this, 1.5 million new cases of PE are identified every year. Moreover, almost 15% of all patients are diagnosed with Malignant Pleural Effusion (MPE) as well.^4^ Patients with lung cancer as advanced stage can be diagnosed with Pleural Effusion (PE).^5^ It is included lots of etiologies classified as benign and malign.^6^ The two most common causes of malignant pleural effusions were lung cancer (37%) and breast cancer (16%).^7^ In Turkey, malignancies (23%–51%) are the leading cause of effusions followed by pneumonia and tuberculosis.^8^ Literature reviews have shown that mortality rates are high in effusions caused by both malignant and benign etiologies.^9^

Computer-based decision support systems have recently been introduced in the field of pleural diseases. Especially in the field of pathology, computer-aided diagnosis systems have been established to detect malignant cancer cells in the cytopathology of pleural effusion and to eliminate the interpretation difference between pathologists. High success has been achieved in “object detection” studies with deep learning methods. In this context, “core sensing” has become a very important field of study in digital pathology.^10^ Studies in which CT images were evaluated using deep learning methods for the diagnosis of tuberculosis pleurisy and malignant pleural mesothelioma are available in the literature.[6,11] There are studies on the differentiation of malignant/benign and transudate/exudate on CT (Hounsfield Unit) of pleural effusions,[11,12] but no study that classifies effusions based on AI has been found in the literature.

This study aims to perform computer-aided numerical analysis of CT images in patients with pleural effusion appearance on CT and to examine its prediction capacity in distinguishing malignant from benign by comparing the quantitative analysis results of the pleural effusion appearance with the cytology results.

### Computer-based decision support systems

The use of computer-aided decision systems in health sciences has developed considerably in recent years.^13^,^14^ Studies on this topic have especially focused on the field of radiological image analysis.^15^ Big data analysis using Artificial Intelligence (AI) and Machine Learning (ML) based classification algorithms in medical science provides an infrastructure for computer-aided diagnosis and clinical decision support systems.^16^ AI has a growing impact on diagnosis systems with the use of deep neural networks to diagnose different diseases, such as lesion/nodules detection, image classification, and image segmentation by pattern recognition.^17^ AI plays a crucial role in the identification of cancer patients by the diagnosis of heterogeneous diseases with an accurate prediction rate. Especially to differentiate between malignant pleural effusion and benign pleural effusion the Deep Learning (DL) technique can be used.^6^ DL is a part of the ML tools that provide high performance in medical imaging, especially for the detection, identification, and segmentation of complex lesions that emerged in image processing and recognition.^18^ Additionally, DL provides highly accurate intelligent diagnosis through medical image analysis.^19^ Whereas computer-aided detection algorithms provide diagnosis systems to assist radiologists by interpreting medical images, DL can learn by extracting effective features from the medical images to differentiate between diseases. As a subfield of ML, DL-based diagnosis models have an increased performance compared to computer-aided detection systems due to improved representations of the lesion features. It helps to improve the additional diagnosis criteria in the diagnosis process by obtaining effective unobservable data from the extracted features from the medical images quantitatively.^20^ DL consists of multiple layers in the optimization process for learning the detected and/or extracted features of lesions/nodules on the related images.^21^ Specifically, DL-based algorithms are trained with CT image data for obtaining the characteristics and diagnostic criteria of related diseases through the extracted/detected features from CT images.^22^

## Methodology

### Diagnostic procedures

While investigating the etiology of pleural effusion, medical treatment is primarily planned in patients with heart failure and minimal pleural effusion, thoracentesis is performed at the initial stage in the other group of patients. Biochemical and cytopathological analysis of the fluid sample by thoracentesis is the major laboratory test to be performed. The separation of exudate/transudate and differentiation of malignant/benign through cytological examination can be made by analyzing the fluid obtained from the pleural space using diagnostic thoracentesis. Light criteria are used to differentiate transudates from exudates.^23^ MPEs constitute 42%–72% of exudative fluids.^1^,^24^ In cases where thoracentesis is not sufficient for the diagnosis, advanced invasive procedures, such as pleural biopsy/video-assisted thoracoscopic biopsy are used in the pathological diagnosis of malignant diseases.

### Imaging methods

Posterior-anterior chest radiography is the common technique used in patients with fluid detection followed by ultrasonography, which provides guidance for thoracentesis. Thoracic ultrasonography helps in characterizing pleural fluid and determining localization while sampling. However, Computed Tomography (CT) is the most preferred method because it gives more information about the nature of the effusion (tumor, if any) and the spread of the disease. In addition to showing the amount and location of the effusion, CT can also detect the presence of diseases such as pneumonia and malignancy underlying the etiology. Moreover, it is very easy to detect and characterize the lesions in CT when contrast material is used. However, imaging findings are not always successful in definitively distinguishing MPEs, mesothelioma, and other diseases. Findings of CT examination have moderate sensitivity and high specificity in distinguishing malign/benign pleural effusion/lesions.^25^,^26^

### Deep learning

Image processing has a crucial role in many medical applications fields.^27^ Deep learning can be used in image processing, image analysis, image classification, image segmentation, feature/object detection, and image retrieval.^28^, ^29^, ^30^ Recently it has provided great success in the detection of nodule regions on medical images and classification of the lymph nodes. Major advances have been provided in the analysis of the sonographic features of nodules on digital medical images for the identification of malignancy by using deep learning.^31^ The core aim of deep learning is to teach computers how to recognize an ideal pattern when giving these approaches a set of data.^32^

### Image-guided decision support for pleural diseases

Pleural disease is becoming more frequent and complex requiring specialist management. Diagnosing and managing the pleural disease can be complex and challenging.^6^ Artificial Intelligence (AI) has been widely used in the field of modern medicine and can help pathologists make more accurate diagnoses.^2^ Additionally, AI in the medical field has become a research hotpot and holds the promise to automatically diagnose heterogeneous diseases with high accuracy.^4^ In recent years, many diagnostic deep-learning approaches such as image classification, disease detection and lesion location have been developed by using chest radiographic images.^33^

One of the main issues in the differential diagnosis of pleural effusion is distinguishing exudates from transudates. Determining the nature of pleural effusion (exudate or transudate) allows for reducing the list of potential pleural causes and indicates the direction for further diagnosis. Another important clinical issue is the etiology of effusion – malignant or benign – is crucial for PE management and prognosis. Combining clinical data using deep learning can enable the development of a novel model for distinguishing the etiology of pleural effusion.^34^

Therefore, developing a useful method that can identify MPE as early as possible with precision is highly important.^35^ Accurate identification of patients with a high probability of MPE is critical to deploy optimal interventions and thus improving patients' clinical outcomes. Hence, a convenient method with a minimum invasion that can accurately identify malignancy from BPE as early as possible is highly desirable. Therefore, it is aimed to explore the applicability of deep learning techniques to distinguish MPE from BPE.^4^

### Proposed method: diagnosis of pleural effusion with deep learning

**Fig. 1:**
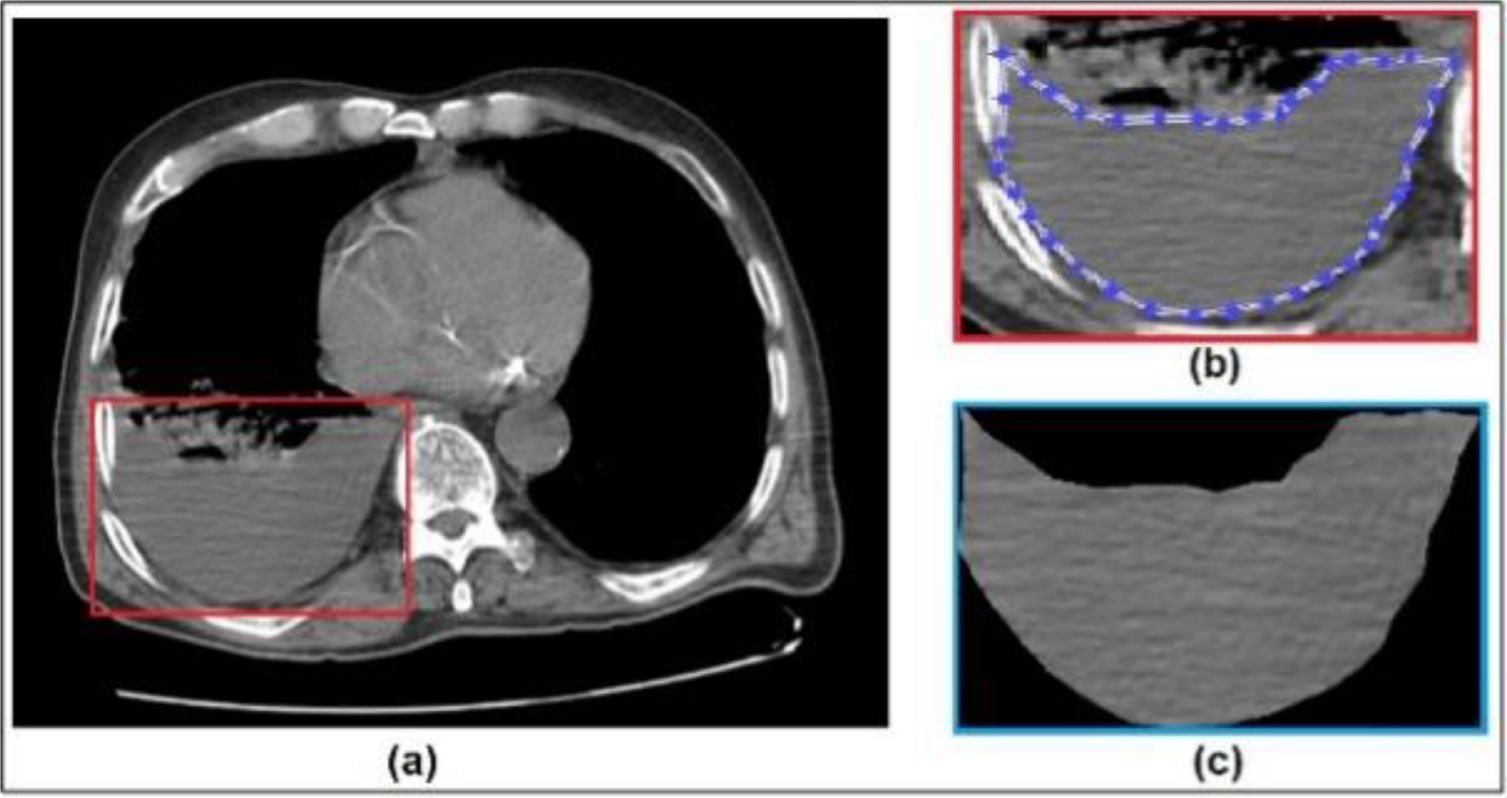
Full size Chest CT image with pleural effusion (1a), region of interest for pleural effusion (1b), extracted feature of pleural effusion (1c).

This study aimed to identify malignant and benign samples from CT images. The general framework of the proposed method is in Figure 1. The texture patches of the PE images were cropped manually by MATLAB (version 9.3.0.713579 [R2017b]). The evaluation process of all cropped PE images was performed by Microsoft Azure Machine Learning Studio-Deep Learning Tool (version 1.6.4.0, release status 3/31/2021) with a license from Recep Tayyip Erdogan University.

The authors conducted this diagnostic study following STARD guidelines.^36^

### Patients

The present study is a retrospective, single-center study using file and image records. Between September 2021 and May 2022, 64 adult patients (> 18 years) patients who were followed up in the clinic with the diagnosis of pleural effusion were included. CT images of the patients were examined, and 408 images of various sections from each patient were recorded in JPEG format. In the next process, the region of interest for pleural effusion is determined and features extracted from chest CT images (Fig. 1). Patient characteristics such as age, sex, weight, height, etiology, and effusion side were collected for further analysis.

### Data characteristics of patients

**Fig. 2:**
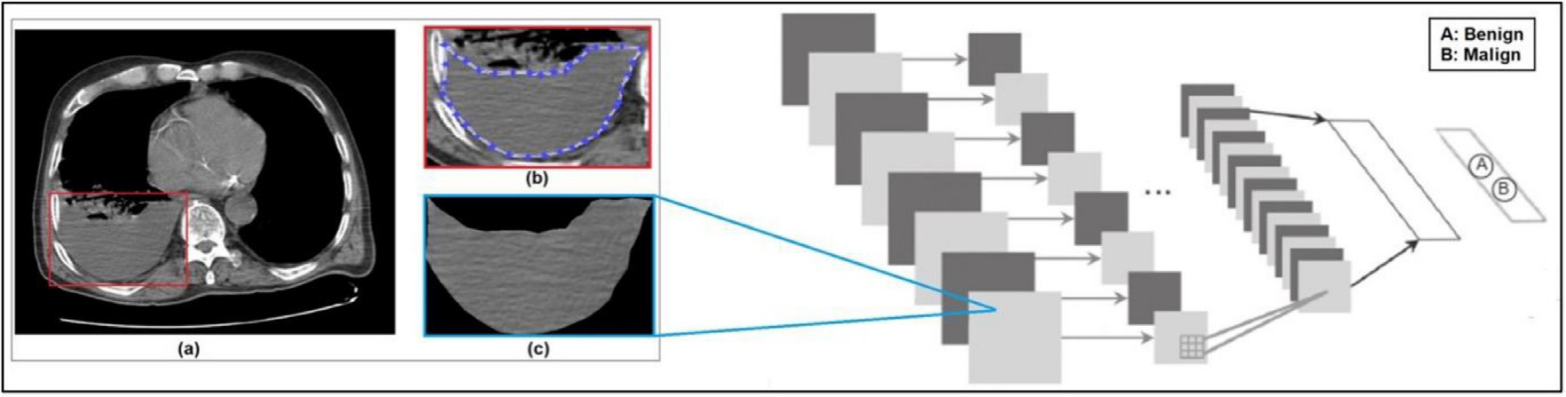
Framework of the deep learning based diagnostic system for pleural effusion (adapted from ^37^).

The cytology results of the thoracentesis fluid samples of the patients were obtained from the file records and were recorded for malignant or benign characteristics. The images obtained were classified by deep learning method within the scope of AI. It used 378 images for the training of the system and different 15 malignant and 15 benign CT images were randomly selected that were used as a test data set. It obtained the prediction output for diagnosis as malign or benign of the tested images (Fig. 2 ‒ Adapted from^37^).

These values obtained by analyzing the diagnostic prediction results of the tested images were compared with the pathology results and the obtained data were evaluated statistically. Diagnostic accuracy, positive predictive value, and negative predictive values were calculated for the computer-aided program to predict the malignant or benign characteristics of the fluid samples.

### Inclusion and exclusion criteria

Patients over 18 years of age who underwent CT with a diagnosis of pleural effusion in the last 5 years were included. Patients with pleural effusion but without thoracentesis indication or who could not undergo thoracentesis due to a contraindication and patients who underwent thoracentesis but did not have cytology examination of thoracentesis fluid were not included in the study. The pathology results of three consecutive thoracenteses were recorded. Especially in cytology results with benign results, three consecutive thoracenteses was determined as an inclusion criterion.

### Statistical analysis

SPSS 17.0 (IBM, Armonk, NY, United States) was used for statistical analysis. Normally distributed data were reported as the mean (±SD), and non-normally distributed data were expressed as the median (interquartile range). The prediction percentages (malignant or benign) of a diagnostic algorithm created with a computer-aided decision system were determined in the tested pleural effusion tomography images. The rates of malignant and non-malignant and benign and non-benign lesions in the malignant and benign effusion tomography images were compared using the Chi-Square test. The receiver operating characteristic curve was constructed to evaluate the diagnostic performance of the computer-aided decision system. Sensitivity, specificity, positive predictive value, negative predictive value, positive likelihood ratio, negative likelihood ratio, and the corresponding 95% Confidence Intervals (95% CIs) were estimated with these cut-off values. A p-value < 0.05 was considered statistically significant.

### Ethics committee approval

The ethics committee approval was obtained from Recep Tayyip Erdogan University Clinical Research Ethics Committee (Ethics Committee Approval no: 2021/90).

## Results

### General characteristics of patients

The mean age of 64 patients (40M, 24F) included in the study was 68 years. The p-values between the training and test sets for age and sex were 0.1307 and 0.3837, respectively, which are greater than 0.05 and indicate a balanced distribution between the two sets (Table 1).

**Table 1:** Demographic, radiological and biochemical characteristics of patients.

	Malign (n = 30)	Benign (n = 34)	p-value
**Mean Age (years)**	70.7 ± 11.1 (46‒97)	66.4 ± 15.5 (20‒95)	0.13
**Gender**			0.38
Male, (n = 40)	16	24	
Female, (n = 24)	14	10	
**Diagnostic Origins of Pleural Effusion**	Adenocarcinoma (n = 13)	Parapneumonic effusion (n = 19)	
	Squamous cell lung cancer (n = 3)	Heart failure (n = 7)	
	Large cell neuroendocrine tumor (n = 1)	Secondary to peritoneal fluid (n = 2)	
	Small cell lung cancer (n = 2)	Tuberculosis (n = 1)	
	Ovarian cancer metastasis (n = 5)	Hypoalbuminemia (n = 2)	
	Prostate cancer metastasis (n = 3)	Renal failure (n = 2)	
	Breast cancer metastasis (n = 2)	Liver failure (n = 1)	
	Renal cell cancer metastasis (n = 1)		

The authors used 378 of the 408 CT images obtained from these patients for training the system, and another 30 CT images for testing the computer system. These 30 images contain 15 malign and 15 benign images for testing the computer system. The training and test sets achieved a balance in most of the characteristics.

### Datasets of the patients

Training Dataset images: 13 patients with lung adenocarcinoma, 5 patients with ovarian cancer metastasis, 3 patients with squamous cell lung cancer, 3 patients with prostate cancer metastasis, 2 patients with breast cancer metastasis, 2 patients with small cell lung cancer, 1 patient with renal cell cancer metastasis, 14 patients with parapneumonic effusion, 7 patients had heart failure, 2 patients had pleural effusion secondary to peritoneal fluid, and 1 patient secondary to tuberculosis.

Test Dataset images: The diagnoses of 15 malignant patients from the patients in the data set used for the test; 7 patients had lung adenocarcinoma, 1 patient had small cell lung cancer, 1 patient had large cell neuroendocrine tumor, and 1 patient had squamous cell carcinoma. And 5 patients had metastatic pleural effusion. Patients with metastases had ovarian cancer metastasis in 2 patients, breast cancer metastasis in 2 patients, and renal cell carcinoma metastasis in 1 patient. The diagnoses of 15 benign patients from the patients in the data set used for the test: parapneumonic effusion in 5 patients, heart failure in 4 patients, hypoalbuminemia in 2 patients, renal failure in 2 patients, liver failure in 1 patient, pleural effusion due to tuberculosis in 1 patient.

### Performance evaluation for the proposed method

**Fig. 3:**
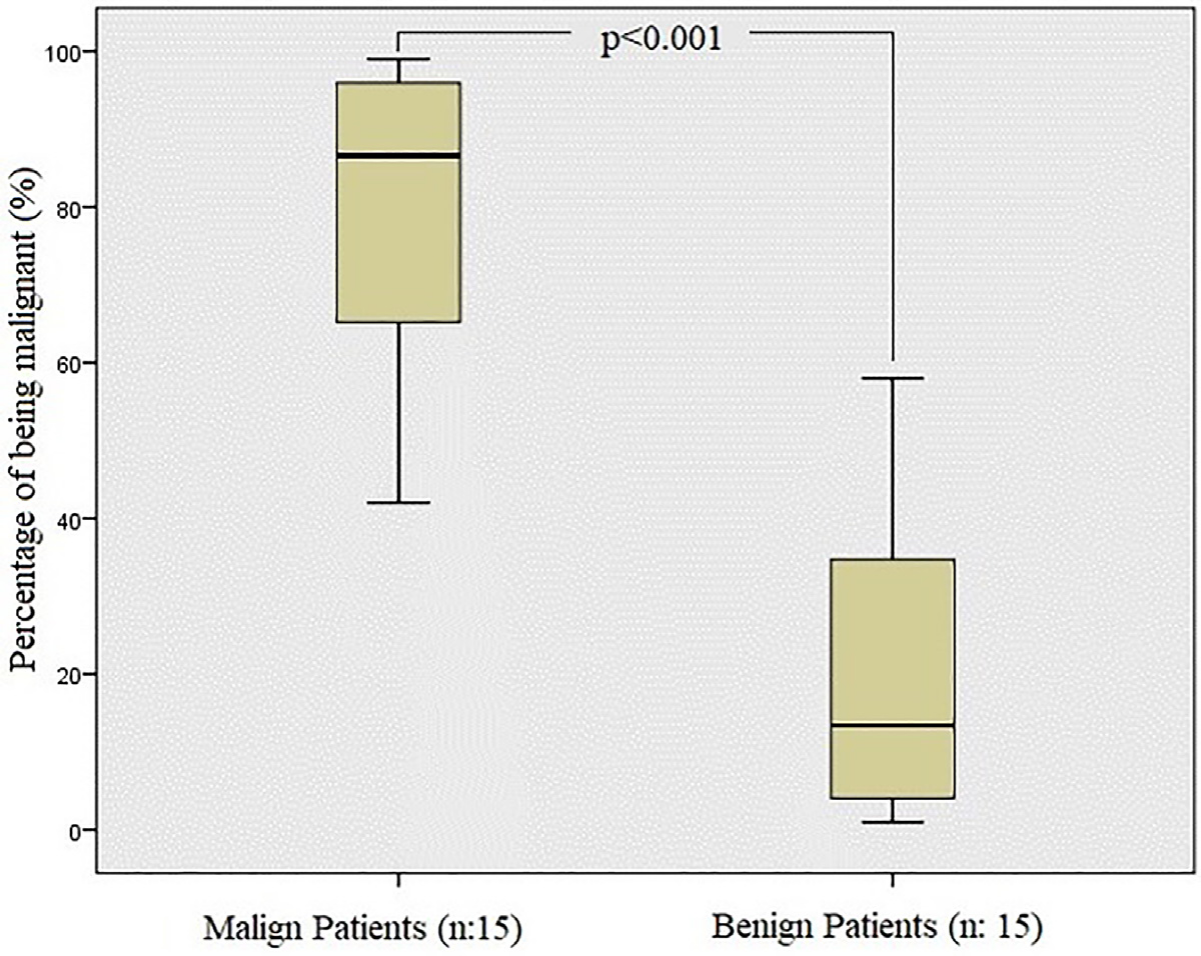
Testing of tomography images of malignant effusions and comparison of predicted diagnosis of malignancy and benign based on the analysis.

**Fig. 4:**
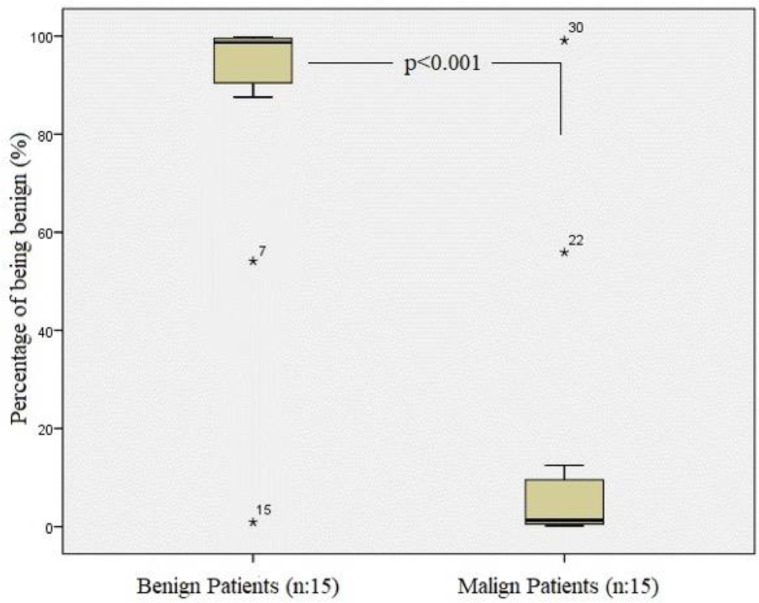
Testing of tomography images of benign effusions and comparison of predicted diagnosis of malignancy and benign based on the analysis.

The diagnosis was correctly predicted in 14 of 15 malignant patients and 13 of 15 benign patients among 30 test images evaluated by the system that is represented as Receiver Operating Characteristics diagnostic yields (PPV: 93.3%, NPV: 86.67%, Sensitivity: 87.5%, Specificity: 92.86%) and statistical significances values (Table 2; Fig. 3, Fig. 4). The diagnostic accuracy calculated by deep learning is 90%.

**Table 2:** Receiver operating characteristics diagnostic yields and statistical significances values.

		95% Confidence Interval	
Performance Metric		Lower Bound	Upper Bound
Sensitivity	0.88	0.617	0.995
Specificity	0.93	0.661	0.998
Positive Predictive Value	0.93	0.667	0.989
Negative Predictive Value	0.87	0.638	0.960
Diagnostic Accuracy	0.90	0.735	0.979
AUC	0.791 (p = 0.007)

**Fig. 5:**
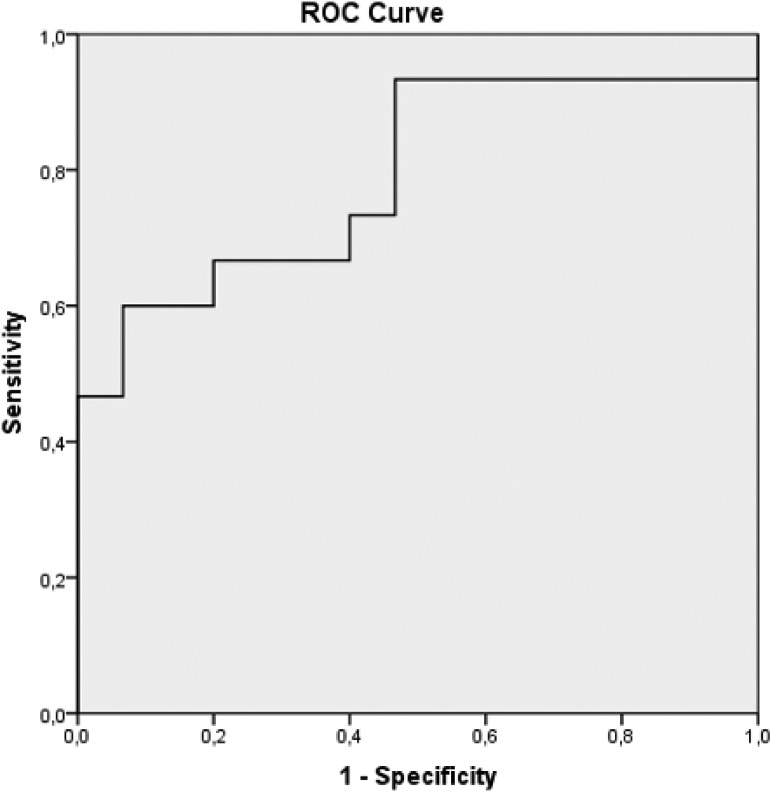
ROC plot with pointwise 95% confidence bounds with the malign and benign pleural effusion CT images train and test with computer-aided decision system. AUC: 0.791; p-value = 0.007.

The model had an Area Under the receiver operating Characteristic Curve (AUC) of 0.791 with a 95% Confidence Interval (95% CI) of 0.623–0.959; p-value = 0.007 (Fig. 5).

## Discussion

Pleural effusion is a condition that may arise from various diseases and affect many people worldwide, but the exact incidence is unknown. Although mortality risk is high in all pleural effusions including malignant or benign, 30-day mortality has been reported as 37% and 1-year mortality as 77% in MPEs.^9^ Therefore, rapid diagnosis of malignancy in patients with pleural effusion is the most critical factor that determines the treatment decision and survival of the patient. If a pleural effusion is detected in a patient, a decision support program that can quickly present a pre-diagnosis, whether malignant or benign, will be an application that meets a great need in this field. Analysis of CT images with deep learning in the present study predicted malignant effusions with a diagnostic accuracy of 90%.

Computer-based decision support systems are now used in many health disciplines to assist physicians and continue to be investigated. There are examples in literature for the usage areas of these applications in pleural diseases and pleural effusions. Computer-aided diagnostic approaches in this area have been studied in various fields since the early 2000s. For example, there are studies using deep learning to automatically detect pleural effusions and pneumothorax in the evaluation of chest radiographs.^38^, ^39^, ^40^, ^41^

Tuberculosis is another disease for which computer-based decision support systems are used in the field of pleural diseases. Seixas et al. has achieved over 90% diagnostic accuracy for tuberculosis diagnosis in adult patients by using an artificial neural networks model containing Human Immunodeficiency Virus positivity and pleural effusion laboratory results (smear, culture, adenosine deaminase, serology, and nucleic acid amplification tests). ANN achieved greater diagnostic accuracy than any individual test considered.^42^ Concerning mesothelioma, which is the primary malignant tumor of the pleura, the focus has been on diagnosis and staging with computer-aided models.^43^ Early-stage pleural mesothelioma is automatically detected by measuring the pleural thickness on CT.^44^ Over time, with the increasing use and experience of AI and DL methods in the field of health, deep convolutional neural networks have come into use for the segmentation of malignant pleural mesothelioma in CT scans.^11^,^21^,^45^,^46^

Computer-based decision support systems are used in the field of pathology to detect MPEs with “core detector” deep learning methods. Win et al. has achieved 87.97% sensitivity, 99.40% specificity, and 98.70% accuracy rate by using an object detector for pleural effusion cytology.^47^

In the literature, there are previous diagnostic predictive studies with the analysis of Positron Emission Tomography (PET) images to differentiate malignant and pleural effusion.^48^ It has been concluded that PET/CT integrated imaging is a more reliable method in distinguishing malignant effusions from benign pleural effusions compared with PET imaging or CT imaging alone. They have also conducted a study to develop an automated system that enables the interpretation of lung ultrasound images. In this study, the data set consisting of 99,209 2D images and 623 videos from 70 patients was interpreted by both the deep learning system and the clinician, and comparable accuracy rates of 92.4% and 91.1% were obtained in the test sets, respectively.^49^ However, to the best of our knowledge, no study has been found in the literature to distinguish between malignant and benign effusions in CT images of the effusions using computer-aided diagnosis algorithms.

The decision-making process of Computer-Aided Diagnosis systems should be explainable to users to obtain their trust in the model. It should be highlighted how computer-based analysis of image features is crucial as more transparent decision-making for diagnosis to final users in healthcare applications.^50^ Computer-based decision support systems as a subset of AI in medicine can become an integral part of the authors’ everyday practice. Therefore, medical specialists, clinical experts, physicians, and medical practitioners should use AI and their role as a decision-support mechanism rather than a competitor.^51^ AI scientific evidence in thoracic imaging has potential clinical utility, implementation and costs, training requirements and validation, its’ effect on the training of new radiologists, post-implementation issues, and medico-legal and ethical issues.^52^

### Limitations of the present study and suggestions for further studies

The most significant limitation of the present study is that it is a single-center and retrospective study. In prospective studies with larger case series, higher diagnostic power, and accuracy can be obtained with better-trained systems by using more test data.

## Conclusion

Advances in computer-aided diagnostic analysis of CT images and obtaining a pre-diagnosis of pleural fluid may reduce the need for interventional procedures by guiding physicians about which patients may have malignancies. The prevalence of such studies and their availability in daily practice can facilitate and accelerate the diagnosis of malignant pleural effusion. Thus, cost and time savings in patient management are achieved in patient management and it can be allowed early diagnosis and treatment.

### Authors’ contributions

(I) Conception and design: NO; (II) Administrative support: AG; (III) Provision of study materials or patients: NO, NMGZ, IS; (IV) Collection and assembly of data: NO, NMGZ, IS; (V) Data analysis and interpretation: AEO, AG; (VI) Manuscript writing: All authors; (VII) Final approval of manuscript: All authors.

## Conflicts of interest

The authors declare no conflicts of interest.
